# Supplementary material for: A scoping review of causal methods enabling predictions under hypothetical interventions
Source: Diagn Progn Res. 2021 Feb 4;5:3. doi: 10.1186/s41512-021-00092-9 (PMC7860039; doi:10.1186/s41512-021-00092-9)
Supplement: Supplementary file 1 — Additional file 1. [file 41512_2021_92_MOESM1_ESM.docx]

# Protocol for “*A scoping review of causal methods enabling predictions under hypothetical interventions*”

## **Overview**

## Prognostic models predict the future occurrence or trajectory of a health outcome based on a set of predictive factors available at the time of prediction. A key limitation of existing prediction models is that they model prognostic risk based on observed risk factors only, and do not allow correct calculation of counterfactual “what if” scenarios to explore the effects of interventions. These “what-if” queries could be enabled within a potential outcomes (counterfactual) framework. Through principled modelling of the underlying causal structure we can infer risk under different intervention scenarios – both at individual patient level and at population level.

*Scope of review*

Modelling interventions in prognostic studies has recently gained attention in clinical prediction model (CPM) research. These interventions typically include medication use, invasive medical interventions (e.g. surgery, invasive measurements), and policy or lifestyle changes that could affect health outcomes. Two categories of treatment: “guided” and “background” were proposed in the review by (Pajouheshnia, Damen, Groenwold, Moons, & Peelen, 2017). Guided treatment, also referred to as “treatment drop-in”, is given to study participants after predictors are measured but before the ascertainment of the outcome. Background treatments are risk-lowering treatments that participants receive as a part of routine care. To date, a dominant approach of research in this area has been on viewing interventions that were applied in the development dataset as potentially biasing (*un-treated*) outcome prediction, and therefore adjusting treatment for effects in the treated individuals. This is termed as ‘passive prediction’ in (Greenland, 2012) in which ‘a treatment is only specially labelled regressor that lacks any formal distinction from other regressors’.

In contrast, we are interested in embedding predictive models in a counterfactual framework -- models that enable predicting the potential outcome of intervention. We want to address questions such as “what would a patient’s risk of having heart attack in 10 years’ time be if he did/did not use statins?”

The objective of our review is twofold: firstly, it will seek to summarise published state-of-the-art in developing counterfactual predictive models; secondly, it will identify potential avenues for future methodological research in this area. We want to clarify assumptions made for each of these methods, report modelling framework, usage, limitations, and available software/packages, and make recommendations for future research. Our target audience are both theoretical researchers and applied statisticians working in clinical healthcare. We aimed to identify published methods for developing and validating prediction models that enable risk estimation of outcomes under hypothetical interventions, utilizing causal inference. We aimed to identify the main methodological approaches, their underlying assumptions, targeted estimands, and potential pitfalls and challenges with using the method. Finally, we aimed to highlight unresolved methodological challenges.

## **Methodology**

## *A known set of articles prior to conducting the review search*

The research team is already aware of a set of published work related to the topic. We group these articles in three categories as in the following. Groups A and B do not offer methodologies that we will be reviewing but include general information to understand and provide insights into this topic. These papers helped us define terms for our search. **Group C (Validation Set) is a set of recent papers that directly address the issue**. These papers will be used to test whether or not our defined search terms are capturing relevant articles.

1. **Review papers clarifying relevant conceptual and practical issues**

Höfler, M. 2005. Causal inference based on counterfactuals. *BMC Medical Research Methodology,* 5**,** 28.

Rubin, D. B. (2005). Causal inference using potential outcomes. *Journal of the American Statistical Association*, *100*(469), 322–331.

Rothman, K. J., & Greenland, S. (2005). Causation and causal inference in epidemiology. *American Journal of Public Health*, *95 Suppl 1*, S144-50.

Clare, P. J., Dobbins, T. A., & Mattick, R. P. (2019). Causal models adjusting for time-varying confounding—a systematic review of the literature. International Journal of Epidemiology, 48(1), 254–265. https://doi.org/10.1093/ije/dyy218

Robins, James M. (1999). Association, Causation, And Marginal Structural Models. *Synthese*. Kluwer Academic Publishers, 121(1/2), pp. 151–179. doi: 10.1023/A:1005285815569.

Robins, J. M., Hernán, M. A., & Brumback, B. (2000). Marginal structural models and causal inference in epidemiology. *Epidemiology (Cambridge, Mass.)*, 11(5), pp. 550–60. Available at: http://www.ncbi.nlm.nih.gov/pubmed/10955408.

1. **Predictive models controlling for interventions, but not necessarily handling predictions under hypothetical interventions**

Alaa, A. M., Yoon, J., Hu, S., & van der Schaar, M. (2018). Personalized Risk Scoring for Critical Care Prognosis Using Mixtures of Gaussian Processes. *IEEE Transactions on Biomedical Engineering*, *65*(1), 207–218.

Groenwold RHH, Moons KGM, Pajouheshnia R, Altman DG, Collins GS, Debray TPA, et al. Explicit inclusion of treatment in prognostic modelling was recommended in observational and randomised settings. J Clin Epidemiol. 2016.

Pajouheshnia R, Peelen LM, Moons KGM, Reitsma JB, Groenwold RHH. Accounting for treatment use when validating a prognostic model: a simulation study. BMC Med Res Methodol. 2017;17(1):103

1. **Predictions under discrete/continuous-time (discrete/continuous-valued) interventions,** including those not explicitly stated as statistical predictive models but indeed developed for the predictive purpose

Sperrin, M., Martin, G. P., Pate, A., Van Staa, T., Peek, N., & Buchan, I. (2018). Using marginal structural models to adjust for treatment drop-in when developing clinical prediction models. *Statistics in Medicine*, *37*(28), 4142–4154.

Schulam, P., & Saria, S. (2017). Reliable Decision Support using Counterfactual Models. Advances in Neural Information Processing Systems 30 (NIPS 2017).

### *Known research groups*

We are aware of several research groups that have published work on methods in related areas. We will therefore manually search for any relevant recent publications from these groups that we may not be aware of prior to conducting the review.

- Miguel Hernan, James Robins, Judith Lok (Harvard T.H. Chan School of Public Health): causality, counterfactuals, longitudinal data
- Suchi Saria (JHU): counterfactual reasoning
- Mihaela van de Schaar (Cambridge): prognosis, diagnosis, treatment
- Michael R. Kosorok (UNCC): individualized treatment
- Susan A. Murphy (Harvard): adaptive interventions in informing clinical decision making

### *Inclusion/Exclusion criteria*

### **Inclusion**

- Papers considering causal effect estimation in the context of health outcome prediction, specifically to enable counterfactual prediction
- Original methodological research (e.g. peer reviewed methodological journal)
- Applied research, which did not develop methodology, but state-of-the-art methodology was employed to address relevant causal prediction questions.

### **Exclusion**

- Non-English articles
- Letters, comments, editorials, and conference abstracts with no information to allow assessment of proposed methods
- Methodology relating to a field other than medical/clinical prediction
- We excluded the studies where the primary objective was calculation of conditional treatment effect, and clinical prediction models were used to infer individualized treatment effects.

### *Information sources*

We will use the above search terms to extract potentially relevant articles from:

- MEDLINE (via Ovid) - **Ovid MEDLINE(R) and Epub Ahead of Print, In-Process & Other Non-Indexed Citations, Daily and Versions**
- Embase (via Ovid)
- Google Scholar
- Non-exhaustive search with textbooks & shallow literature search on broad causal inference methods

*Search Terms*

We have known of common methods in causal inference from prior knowledge. These terms were extracted from textbooks and known relevant literatures and we used them for keyword search in the databases. Grey terms were included in the initial attempt but removed from the final search as the resulted amount was not manageable often due to the broadness of the terms. We notice that papers from the validation set were still found after removing these broad terms.

| **Table 1. Causal methods: common methods used for causal inference** |  |
| --- | --- |
| MEDLINE mesh search for ‘models’:  models, statistical/ or logistic models/ or *models,theoretical/ or likelihood functions/ or survival analysis/  EMBASE mesh search for ‘models’:  statistical model/ or *analytic method/ or statistical analysis/ or regression analysis/ or logistic regression analysis/ or maximum likelihood method/ or *theoretical model/ or *survival/ or *survival rate/ | 290496  704509 |
| (causal effects or causal graphical or causal inference) adj3 (method$ or model$) | 326 |
| ~~structural equation model*~~ | ~~32412~~ |
| structural equation model*.ti,ab. | 15108 |
| structural model*.ti,ab. | 25765 |
| structural nested model* | 68 |
| structural nested mean model* | 20 |
| marginal structural model* | 1467 |
| sufficient-cause model* | 17 |
| counterfactual model* | 59 |
| g-estimation OR g-computation OR g-formula OR g-method$ | 549 |
| inverse probability weight* OR inverse probability of treatment OR IPTW | 2877 |
| (stratification ~~OR restriction~~ OR matching OR standardi$ation)  ~~AND causal~~ | 5542 |
| (propensity score$ OR propensity model$) ~~AND causal~~ | 51394 |
| instrument* variable* | 5257 |
| causal forest* | 7 |
| double robust estimat* | 73 |
| targeted maximum likelihood OR TLME | 128 |
| Time-dependent confounding OR Time-dependent confounder$ | 297 |
| time-varying treatments OR time-varying confounding OR time-varying confounder$ OR treatment-confounder | 295 |
| Treatment-confounder feedback | 1 |
| ~~adjust* adj1 confound*~~ | ~~2721~~ |

We filter results from Search 1 using two filters.

### **Filter 1: Causal framework**

This filter is to search for general articles within the scope of causality and causal inference.

| **Table 2. Causal Aspect Filters: general papers on causal inference and potential outcome (counterfactual) framework; all combined using OR operation** |  |
| --- | --- |
| **MEDLINE mesh:**  causality/ OR precipitating factors/ OR "confounding factors (epidemiology)"/ OR *epidemiologic methods/ OR epidemiologic factors/ OR exp effect modifier, epidemiologic/  **Embase mesh:**  *epidemiology/ or *confounding variable/ or *epidemiologic data/ or *causal attributes/ or causal modeling/ or "causal inference test"/ | 3184217  45060 |
| (causalit*).ti,ab. OR causal inference OR causal effect* OR causation OR causal model* | 72158 |
| ~~causalit*~~ | ~~50408~~ |
| ~~causalit*.ti,ab.~~ | ~~14610~~ |
| counterfactual* | 3051 |
| potential outcome* | 3631 |
| what if reasoning | 1 |
| causal intervention$ | 106 |
| treatment$ intervention$ | 4137 |
| clinical intervention$ | 7141 |
| treatment drop-in | 10 |
| ~~treatment effect*~~ | ~~83808~~ |

Note that we are interested in building rigorous clinical predictive models. A series of search strings specifically designed for use in reviews of clinical prediction research were proposed by Geersing *et al.* *Geersing string* is based on the search terms proposed by Ingui et al. and includes an additional update which has been shown to provide excellent sensitivity in picking up articles related to clinical prediction model development. We therefore anticipate that it should provide a comprehensive means of finding papers related to methodological development in CPMs and will use them as filtering terms combined (AND) with Search 1 results.

### **Filter 2: Clinical predictive models filter**

| **Table 3. CPM FILTER:** Ingui CPM Search Strategy (Rows 1-5) + Geersing Update (Row 6) |
| --- |
| (Validat$ OR Predict$.ti. OR Rule$) |
| (Predict$ AND (Outcome$ OR Risk$ OR Model$)) |
| ((History OR Variable$ OR Criteria OR Scor$ OR Characteristic$ OR Finding$ OR Factor$) AND (Predict$ OR Model$ OR Decision$ OR Identif$ OR Prognos$)) |
| (Decision$ AND (Model$ OR Clinical$ OR Logistic Models)) |
| (Prognostic AND (History OR Variable$ OR Criteria OR Scor$ OR Characteristic$ OR Finding$ OR Factor$ OR Model$)) |
| Stratification OR ROC Curve OR Discrimination OR Discriminate OR c-statistic OR c statistic OR Area under the curve OR AUC OR Calibration OR ~~Indices~~ OR Algorithm OR Multivariable |

# Extended Results

## E.1 Combining causal effects measured from external information

Three papers (Brunner et al., 2019; Candido dos Reis et al., 2017; Silva, 2016) were identified as developing models with combined information from different sources to address single treatment effect. Candido dos Reis *et al.* (Candido dos Reis et al., 2017) and Brunner *et al.* (Brunner et al., 2019) took a two-stage approach, in which causal effect estimates from external sources such as RCTs and meta-analyses were first identified, then combined with prediction models to allow counterfactual predictions.

In Candido dos Reis *et al.* (Candido dos Reis et al., 2017), a CPM was developed to predict mortality from breast cancer. A treatment variable (adjuvant chemotherapy and adjuvant hormone therapy) was added to the prediction model as a predictor, and the ‘treatment benefits’ (coefficients) were fixed to the effect sizes reported from a meta-analyses of 194 randomised trials (Abe et al., 2005). Brunner *et al.* (Brunner et al., 2019) developed a CPM for cardiovascular risk which was then combined with an externally estimated risk reduction model to aid decision making in lipid-lowering treatment usage. The risk reduction model was estimated from meta-analyses with genetic studies, prospective epidemiologic studies, Mendelian randomization studies, and RCTs to obtain an equation for the expected average proportional risk reduction per mmol/L reduction in LDL cholesterol (Ference et al., 2017). To calculate the treatment effect on cardiovascular risk, the approach assumed a reduction of baseline non­HDL cholesterol by 30% or 50% when lipid-lowering treatment was used.

In addition to the above two-stage approach borrowing causal information estimated externally into predictive models, a one-stage approach was also identified where the two sources of data, interventional and observational, were jointly modelled for causal prediction (Silva, 2016). This approach was applied in a scenario where it is possible to collect interventional data such that treatments were controlled but where sample sizes might be limited. The idea was to transform observational data into informed priors under a Bayesian framework. Assume that the observational data $D_{obs}$ of size $N$ and the interventional data $D_{int}$ of size $M$were provided, and the measure variables $X$ were assumed to satisfy the *back-door criteria* (Pearl, 2011). Their goal was to predict the *dose-response curve* under intervention, or “dose” $a$: $f\left( a \right)\equiv E [Y^{\left( a \right)}]$ where $a\in\boldsymbol{A}$, a pre-defined set of treatment levels, and $Y^{(a)}$ is the potential outcome $Y$ under the intervention $a.$ Define the conditional mean function$g\left( a,x \right)=E \left[ Y | A=a, X=x \right]$ over the observational input space $\left( \boldsymbol{A,X} \right)\boldsymbol{,}$ and the adjusted observational dose-response curve as $f_{obs}\left( a \right)=\int g(a,x)p(x)dx$. Silva (Silva, 2016) assigned a two-layered prior to $f(\cdot)$ in a observational-interventional setup as follows:

$$f_{obs}\left( \boldsymbol{A} \right)\sim\mathcal{GP}\left( \mu_{obs},K_{obs} \right),$$

$$f\left( \boldsymbol{A} \right) = \alpha\left( \boldsymbol{A} \right)\odot f_{obs}\left( \boldsymbol{A} \right)+\beta\left( \boldsymbol{A} \right),$$

$$Y_{int}^{i} \sim\mathcal{N}\left( f\left( a_{int}^{i} \right),\sigma_{int}^{2} \right), 1\leq i\leq M.$$

Here, a Gaussian process prior over adjusted dose-response curves was first estimated from observational data, and then composed with an “element-wise affine” transformation estimated from interventional data to bias $f$ toward $f_{obs}$. The prediction can then be made by inferring the posterior distribution of the dose-response curve $f(\boldsymbol{A})$ or the individual posterior distribution over $f_{obs}(\boldsymbol{A})$; conditional dose-response can also be estimated from $f(\boldsymbol{A})$ and $f_{obs}(\boldsymbol{A})$ conditioned on a subset of covariates $S\subset X$.

All approaches discussed in this section are limited to a single intervention type and intervening at a single point in time. Approaches that directly apply the causal effects that have been estimated externally into CPMs assume that the estimated causal effects are generalisable to the population in which one wishes to apply the prediction model. Equally, combining individual data from both sources (i.e. the one-stage approach) ignored the issue of sample selection bias, which was highlighted in (Silva, 2016). Additionally, the one-stage approach can become computationally intensive since the cost of computing the covariance matrix $K_{obs}$, $\mathcal{O(}T^{2}N^{2})$, is potentially high, as the size of the observational data ($N$) and number of treatment levels ($T$) increases.

## E.2 Estimating both a prediction model and causal effects from observational data

A total of 9 papers discussed modelling counterfactual predictions entirely from observational data. Approaches from these papers can be further divided into two categories: (1) methods considering only one intervention at a single time point (Alaa & van der Schaar, 2017; Arjas, 2014; van Amsterdam, Verhoeff, de Jong, Leiner, & Eijkemans, 2019), as discussed in the following section E.2.1, and (2) methods allowing time-dependent interventions (Bica, Alaa, Jordon, & van der Schaar, 2020; Lim, 2018; Schulam & Saria, 2017; Soleimani, Subbaswamy, & Saria, 2017; Sperrin et al., 2018; Xu, Xu, & Saria, 2016), as discussed in section E.2.2. Throughout the rest of this paper, we will use the following notations to illustrate the methods covered.

##### Notations

For sequential outcomes and longitudinal data settings, we have repeated observations of outcome $\boldsymbol{Y}_{\boldsymbol{i}}=\{Y_{ij}: j=1, \ldots, J_{i}\}$ from the$i$th individual at (not necessarily regularly-sampled) times $\{t_{i1},\ldots, t_{iJ_{i}}\}$. The outcome $Y_{ij}$ can be any data type (binary, continuous, time-to-event, count data, etc.). In addition, we have $\boldsymbol{X}_{\boldsymbol{i}}=\{X_{ij}:j=1,\ldots,K_{i}\}$, where $X_{ij}$ is a 1 by p vector of observed covariates (e.g. age, gender) about this individual, which may or may not be time-varying. We also have interventions $\boldsymbol{A}_{\boldsymbol{i}}=\{A_{il}: l=1, \ldots, L_{i}\}$ that were applied to patient $i$ at times $\{\tau_{i1},\ldots, \tau_{iL_{i}}\}$, where $A_{il}=a$ for either a value $a\mathbb{\in R}$ such as treatment dose, or some intervention type $a\in\{a_{1}, \ldots, a_{D}\}$. Note that the time for interventions $\tau_{il}$ and time for outcome measurements $t_{il}$ need not be the same. The potential outcome under an intervention $a$ is denoted as $Y^{(a)}$. In the continuous-time setting, we use the subscript $t$ to indicate measurements taken at time $t$. The value of a measurement $\boldsymbol{X}_{\boldsymbol{i}}$ up to time $t$ exclusive is denoted by $\boldsymbol{X}_{\boldsymbol{i,<t}}$. We use the same notation for settings where one or more of $\boldsymbol{Y, X}$, and $\boldsymbol{A}$ might only be measured once, in which case we restrict the corresponding respective $J$,$K$, and/or $L$ to be 1. When there is no ambiguity, subscripts of notations may be omitted where convenient, depending on the context.

### E.2.1 Counterfactual prediction models that consider an intervention at a single point in time

***Related to average treatment effects***

In a scenario where there is a clinical need for outcome prediction and the individual outcome partly depends on treatments, one requires an unbiased estimate of the treatment effect at baseline to decide whether to intervene on treatment. Assuming that the Directed Acyclic Graph (DAG) that encodes the relationship between all the relevant variables is known, then *do-calculus* (Pearl, 2011) provides an indication of whether this can be achieved in the setting of observational data with the required causal assumptions. Van Amsterdam *et al.* (van Amsterdam et al., 2019) addressed a particular scenario where the prognostic factors (CT-scans of lung cancer) also contained information of colliders (e.g. tumor size and heterogeneity) that appeared on the DAG for estimating treatment effects on the survival. The authors proposed a multi-task prediction scheme embedded in a convolutional neural network (CNN) framework for both the outcome and the collider. The collider tumor size $x$, cannot be directly observed but can be measured from the image. Given the survival time, treatment, and scan image for each patient, a CNN was trained to predict survival and estimate treatment effect. Following standard practice for predicting a continuous real outcome with deep learning, the last layer of the CNN resembles linear regression where

$$\hat{Y}=\beta_{0}+\beta_{a}a+ \sum_{j=1}^{N_{k}} \beta_{j}^{k}\phi_{j}^{k}$$

with $\phi_{j}^{k}$ being the $N_{k}$ activations of the final layer of a $k$-layer CNN and $\beta_{a}$ estimating the average treatment effect. The idea was to utilise this resemblance and separate the contributions of different factors of variation during training. To attain separation of the collider $x$ from other factors of variation in the last layer, a loss term ${L_{x}(\phi}_{1}^{k},x)$ was added to the total loss function such that a single activation will approximate the collider: $\phi_{1}^{k}\approx x$. Meanwhile, the other activations were constrained to be linearly independent of the collider so that performing linear regression on these activations ($\phi_{j}^{k}, j>1$) and the treatment indicator $a$should mimic ommitting the collider as a variable in the regression. After model convergence, one can fix all CNN parameters and do a single ordinary least squares on $\{\phi_{j}^{k}\cup a, j>1\}$to get a valid estimate of the treatment effect with $\beta_{a}$ and make prediction on the overall survival.

Van Amsterdam *et al.* (van Amsterdam et al., 2019) has demonstrated that deep learning can in principal be combined with insights from causal inference to estimate unbiased treatment effect for prediction. However, the causal structure applied here was in its simplest form, and further developments are needed for more realistic clinical scenarios where, e.g., there is confounding for treatment assignment, or a treatment effect modifier exists within the image.

***Related to conditional treatment effects***

Conditional treatment effects for subjects with a covariate $X=x$ in a population at a single time point is defined as $T\left( x \right)=E[Y^{\left( 1 \right)}-Y^{\left( 0 \right)}|X=x]$ and our goal here is to estimate the counterfactual prediction of $E\left[ Y^{\left( a \right)} | X=x \right], a\in\{0,1\}$. In an RCT, given complete randomisation, i.e. $a$ is independent of $Y^{\left( a \right)}$ and $X$, under consistency, one can estimate $E\left[ Y^{\left( a \right)} | X=x \right]$ by fitting a prediction model to the treated arm ($a=1$) and the control arm $(a=0)$, respectively. The technique is often used in estimating conditional treatment effects (Li et al., 2016; Nguyen, Collins, Landais, & Le Manach, 2020) or identifying subgroups from RCTs (Cai, Tian, Wong, & Wei, 2016; Lamont et al., 2018), whereas our focus is counterfactual prediction under interventions. In Alaa *et al.* (Alaa & van der Schaar, 2017)*,* under a set of assumptions, this technique was adapted for counterfactual prediction with observational data, which, nevertheless, used a more complex regression model to address for selection bias in the observational dataset.

Alaa *et al.* (Alaa & van der Schaar, 2017) adopted standard assumptions of *unconfoundedness* (or *ignorability*) and *overlap (or positivity)*, which is known as the "potential outcomes model with unconfoundedness". They used the signal-in-white-noise model for the potential outcomes $Y_{i}^{\left( a \right)}$, such that,

$$Y_{i}^{\left( a \right)}=f_{a}\left( X_{i} \right)+\epsilon_{i,a}, a\in\{0,1\},$$

where $\epsilon_{i,a}\mathcal{\sim N}\left( 0,\sigma_{a}^{2} \right)$, and the conditional treatment effects can then be estimated as $\hat{T}\left( x \right)=\hat{f}_{1}\left( x \right)-\hat{f}_{0}\left( x \right)$. The two target functions $f_{0}\left( \cdot\right)$ and $f_{1}\left( \cdot\right)$ were estimated simultaneously with training data through one loss function, known as the precision in estimating heterogeneous effects (PEHE), which jointly minimises the error of factual outcomes and the posterior counterfactual variance. The regularised empirical PEHE minimisation was shown to be equivalent to Bayesian inference with a multi-task Gaussian process prior on the vector-valued potential outcomes function ${f\left( \cdot\right)= {[f}_{0}\left( \cdot\right){, f}_{1}\left( \cdot\right)]}^{T}:$ $f\sim\mathcal{GP}(0,K_{\theta})$. This equivalence allowed reasoning about the unobserved counterfactual outcomes in a Bayesian fashion. The potential outcomes function $\hat{f}$ can then be estimated from the posterior mean of the model, followed by the potential outcome prediction $\hat{Y}^{(a)}$. Since the ground truth counterfactual outcomes are never available in real-world observational datasets, it is not straightforward to evaluate causal prediction algorithms and compare their performances, a semi-synthetic experimental setup was adopted in (Alaa & van der Schaar, 2017), where covariates and treatment assignments are real but outcomes are simulated.

For the longitudinal setting where the event history is fully observed, Arjas (Arjas, 2014) adopted a marked point process (MPP) framework to predict a final outcome under a single intervention. In a Bayesian non-parametric hazard model for the final outcome occurrence, past measurements and treatments were wrapped up to form a “internal history” for each individual, and then a continuous-time outcome variable intensity function $\lambda_{i}(t)$ relative to the histories was specified for each individual $i$. The likelihood contribution from the outcome events of the individual $i$ was then of the standard Poisson form $\prod_{j} \lambda_{i}\left( t_{ij} \right)\text{exp} \{-\int\lambda_{i}\left( s \right)ds\}$. The statistical inference can be made by embedding all observed events in the data in the form of marked points into a single MPP under the assumptions of *local independence* and *conditional independence*, which can be seen as postulating a dynamic version of *no unobserved confounders* and *exchangeability*, respectively. Counterfactual queries can be then answered by evaluating the corresponding predictive probabilities based on the available data.

Both methods in this subsection can be computationally intensive as the number of observed samples increased. This could be ameliorated using conventional sparse approximations (Alaa & van der Schaar, 2017; Rasmussen & Williams, 2006). Both methods are limited to binary interventions, and prediction via treatment effect estimation can only make counterfactual prediction for outcomes with or without intervention.

### E.2.2 Counterfactual prediction models that consider time-dependent treatments and treatment-confounder feedback

Papers included in this category (Bica et al., 2020; Lim, 2018; Schulam & Saria, 2017; Soleimani et al., 2017; Sperrin et al., 2018; Xu et al., 2016) covered three types of approaches to deal with scenarios where the treatments of interest and confounders vary over time. One example of such confounding is in the sequential-treatment assignment setting, where doctors use a set of variable measurements, at the current time or in the past, to determine whether or not to treat, which in turn affects values of these variables at a subsequent time. The assumptions needed for identifying unbiased treatment effects in such scenarios are *consistency*, *positivity*, and *sequential ignorability*.

***Marginal structure models (MSMs) within a prediction model framework***

MSMs with inverse probability treatment weighting (IPTW) for receipt of treatment is a common way to estimate the unbiased treatment effects where a pseudo-population is created such that treatment selection will be unconfounded. Motivated by estimating treatment-naïve risk in presence of *treatment drop-ins*, i.e., treatments initiated post baseline, Sperrin *et al.* (Sperrin et al., 2018) proposed combining MSM with predictive modelling approaches to adjust for confounding and generate prediction models that could appropriately estimate risk under the required treatment regimens. Following the classic development of IPTW for an MSM, the model first calculated weights using the post baseline treatment history $\bar{A},$and baseline variables $X_{0}$ comprising all variables that are prognostic for $Y$ (rather than only the effect modifiers). For outcome prediction, it then fitted a logistic model with the derived weights

$\text{logit} P\left[ Y=1 \right|X_{0}, \bar{A}]= \beta_{0}+ \beta_{X}X_{0}+\sum_{k=0}^{K} {( \beta}_{A_{k}}A_{k}+ \beta_{A_{k}X}A_{k}X_{0})$ .

Estimating treatment-naïve risk, which corresponds to the causal estimand $E\left[ Y^{(\bar{A}=0)} \right|X_{0}]$, can truly support guiding treatment initiation. By carefully defining the required estimand for the target prediction, the proposed framework could estimate risks under a variety of treatment regimens. As with approaches described so far in this category, the model only considered a binary treatment. The extension to multiple treatment choices for the proposed method is possible in principal; although, the underlying causal structure and resulted model may become too complex.

Similarly to (Sperrin et al., 2018), Lim *et al.* (Lim, 2018) adopted the MSM combined with IPTW approach. Instead of using linear or logistic regression models, they embedded the concept into a deep learning framework and proposed a *Recurrent Marginal Structural Network* (RMSN). The model consisted of (1) a set propensity networks to compute treatment probabilities used for IPTW, and (2) a prediction network used to determine the treatment response for a given set of planned interventions. In propensity networks, the key probability functions $p\left( A_{t} | \bar{X}_{t-1} \right)$ and $p\left( A_{t} | \bar{A}_{t-1},\bar{X}_{t} \right)$ were parameterized with different long short-term memory (LSTM) models, $\mathrm{RNN}_{\mathrm{SW}_{1}}\left( \bar{X}_{t-1} \right)$ and $\mathrm{RNN}_{\mathrm{SW}_{2}}\left( \bar{A}_{t-1}, \bar{X}_{t} \right)$, respectively. In the prediction network, RMSN used an encoder network (a standard LSTM) to predict one-step-ahead outcome $Y_{t+1}$ given observations of covariates and actual treatments, and to generate the internal state $h_{t}$ of the LSTM

$$g\left( A_{t},\bar{X}_{t} \right)=\mathrm{RNN}_{E}\left( \bar{A}_{t},\bar{X}_{t} \right), h_{t}=\mathrm{RNN}_{\hat{E}}\left( \bar{A}_{t},\bar{X}_{t} \right).$$

RMSN then used a decoder network to propagate the encoder representation $h_{t}$ forwards in time and estimate the treatment responses for a sequence of planned treatments in the future

$$g\left( a_{t+1},\ldots,a_{t+\tau}{, \bar{X}}_{t} \right)= \mathrm{RNN}_{D}\left( h_{t}, a_{t+1},\ldots,a_{t+\tau}{, \bar{X}}_{t} \right), \forall\tau>1.$$

Stabilised weights were used to weight the loss contributions of each training observation in both encoder and decoder network. Details of the training procedure are illustrated in (Lim, 2018).

The benefit of RMSN is that, it can be configured to have multiple treatment choices and outcomes of different forms (e.g. continuous or discrete) using multi-input/multi-output RNNs. Treatment sequences can also be evaluated and no restrictions were imposed on the prediction horizon or number of planned interventions. The use of LTSMs in computing the probabilities required for propensity weighting can also alleviate susceptibility of IPTWs to model misspecification. A drawback is that one needs a rich source of longitudinal data to train the model. Moreover, as in general in deep learning models, they lack a clear interpretation.

***Methods based on balanced representation approach***

Matching approaches such as MSM or RMSN combined with IPTW above adjust for bias in the treatment assignments by creating a pseudo-population where the probability of treatment assignments does not depend on the time-varying confounders. Balanced representation approach, as proposed by Bica *et al.* (Bica et al., 2020), instead aimed for a representation $\Phi$ of the patient history $\bar{H}_{t}=\left( \bar{A}_{t-1},\bar{X}_{t} \right)$ that was not predictive of treatment assignments, i.e.,

$$P\left( \Phi\left( \bar{H}_{t} \right) | A_{t}=a_{1} \right)=\cdots=P\left( \Phi\left( \bar{H}_{t} \right) | A_{t}=a_{D} \right),$$

where $\{a_{1},\ldots, a_{D}\}$ are $D$ possible treatment assignents at time $t$. It can be shown that, in this way, estimation of counterfactual treatment outcomes is unbiased (Robins, 1999). Bica *et al.* (Bica et al., 2020) proposed a counterfactual recurrent network (CRN) to achieve balancing representation and estimate unbiased counterfactual outcomes under a planned sequence of treatments. CRN encoder used domain adversarial training to handle the bias from the time-dependent confounders where the different treatment choices at each timestep were considered the different domains, and built a treatment invariant representation $\Phi\left( \bar{H}_{t} \right)$. The decoder network then used $\Phi\left( \bar{H}_{t} \right)$ to initialise the state of an RNN that predicted the counterfactual outcomes for a sequence of future treatments.

CRN improved the closely related RMSN model proposed by Lim *et al.* (Lim, 2018) in a way that overcame the fundamental problem with IPTW, such as the high variance of the weights. As with RMSN, both models required hyperparameter tuning. As the counterfactual outcomes were never observed, hyperparameters in both models were optimised based on the error on the factual outcomes in the validation dataset. As noted by the authors in (Bica et al., 2020), more work on providing theoretical guarantees for the error on the counterfactuals are required.

***Methods with g-computation for correcting time-varying confounding***

Three papers (Schulam & Saria, 2017; Soleimani et al., 2017; Xu et al., 2016) were identified using g-computation to correct time-varying confounding and predicting treatment response curves under the potential outcome framework.

Xu *et al.* (Xu et al., 2016) developed a Bayesian non-parametric model for estimating conditional treatment response curves under the g-computation formula, and provided posterior inference over the treatment response curves. It modelled the potential outcome $Y_{ij}$ using a generalized mixed-effects model combining the baseline progression (with no treatment prescribed), the treatment responses overtime, and noise:

$$Y_{ij}|X_{ij},A_{i,<t_{ij}}=\underset{\text{baseline progression}}{\underbrace{b\left( X_{ij} \right)+ u_{i}\left( t_{ij} \right)}}+ \underset{\text{treatment response}}{\underbrace{f_{i}\left( t_{ij};A_{i,<t_{ij}} \right)}}+ \underset{\text{noise}}{\underbrace{\epsilon_{i}\left( t_{ij};A_{i,<t_{ij}} \right)}}.$$

The goal was to obtain posterior inference for the treatment response, and predict the potential outcomes $Y_{i,>t}$ given any sequence of treatments conditioned upon past treatments and covariate history. The additive treatment effects over time was modelled with a function of treatment duration for each individual $f_{i}\left( t_{ij};A_{i,<t_{ij}} \right)= \sum_{l:\tau_{il}<t_{ij}} g_{i,A_{il}}(t_{ij}-\tau_{il})$, where $g\left( t \right)$ was carefully chosen by concatenating two sigmoid curves to flexibly capture responses for a duration over which the treatment took effect. To capture individual heterogeneity, a nonparametric hierarchical prior was imposed on the parameters in the baseline progression and the treatment response model using a Dirichlet process (DP) mixture. There are two limitations to the model here: (1) it assumes independent baseline progression and treatment response components; (2) treatment response models rely on strong assumptions and clinical details to be decided by domain experts.

Soleimani *et al.* (Soleimani et al., 2017) extended the approach in Xu *et al.* (Xu et al., 2016) in two ways: (1) to continuous-time setting with continuous-valued treatments, and (2) to multivariate outcomes. Similarly to (Xu et al., 2016), for each individual $i$ and outcome type $d$, the outcome trajectory $Y_{id}(t)$ was modelled with three parts: treatment response, fixed effects for baseline progression, and random effects to capture correlations within and across outcomes. For the treatment response component, the model used differential equations--linear time-invariant dynamical systems (LTI)-- to capture the relationship between the response curve and treatment-dose curve and therefore allow for modelling the treatment response curves under continuous changes in dose level over time. Finally, by decomposing each of the three parts of the model into a shared and an outcome-specific component, the model allowed for jointly modelling all outcome variables for each individual. Despite being a more flexible model than (Xu et al., 2016), this model did not overcome two limitations mentioned above.

Schulam and Saria (Schulam & Saria, 2017) considered another continuous-time setting where both type and timing of actions may be dependent on the preceding outcome, and one needs to predict how a continuous-time trajectory will progress under sequences of actions. The goal was to model action-outcome traces $D\equiv\left\{ t_{ij},Y_{ij},a_{ij} \right\}_{i, j}$for individual $i$ with irregularly sampled sequences of actions and outcomes. Schulam and Saria (Schulam & Saria, 2017) proposed a Counterfactual Gaussian process (CGP) model to model the trajectory and derived an adjusted maximum likelihood objective that learned the CGP from observational traces. The objective was derived by jointly modeling observed actions and outcomes using a marked point process (MPP), with MPP intensity as a product of three models

$$\lambda^{*}\left( t,Y,a,z_{Y},z_{a} \right)=\underset{\text{Event model}}{\underbrace{\lambda^{*}\left( t \right)p^{*}\left( z_{Y}, z_{a} | t \right)}}\underset{\text{Outcome model (GP)}}{\underbrace{p^{*}\left( Y|t,z_{Y} \right)}}\underset{\text{Action model}}{\underbrace{p^{*}\left( a|Y, t,z_{a} \right)}},$$

where the *∗* superscript indicates that the above hazard function and densities are implicitly conditioned on the history of previous observations of the process and all previous actions. Note here that $t$ denoted both action and outcome measurement time, and that binary random variables $z_{Y}, z_{a}\in\{0,1\}$ to indicate whether it was the outcome or the action was observed and the unobserved variable was assigned a null value $\emptyset$. The outcome model was parametrised using a Gaussian process (GP), or a hierarchical GP or mixture of GPs to capture the individual heterogeneity, and can be treated as a standard regression model that predicted the trajectory progression given the previous actions and outcome observations. The potential outcome query can therefore be answered with the posterior predictive trajectory of the outcome model. A key limitation in this model is that it could not model heterogeneous treatment effects arising from baseline variables.

Counterfactual prediction models in this section using g-formula to correct for time-varying confounding are highly flexible and can be adopted for a variety of clinical settings. However, these methods rely on a set of strong assumptions in both discrete-time and continuous-time settings that are generally not testable; for the latter, Schulam and Saria (Schulam & Saria, 2017) extended Robin’s *Sequential No Unobserved Confounders* assumption to continuous-time case and also assumed *Non-informative Measurement Times*.

# Reference:

Abe, O., Abe, R., Enomoto, K., Kikuchi, K., Koyama, H., Masuda, H., … Caffier, H. (2005). Effects of chemotherapy and hormonal therapy for early breast cancer on recurrence and 15-year survival: An overview of the randomised trials. *Lancet*, *365*(9472), 1687–1717. https://doi.org/10.1016/S0140-6736(05)66544-0

Alaa, A. M., & van der Schaar, M. (2017). Bayesian Inference of Individualized Treatment Effects using Multi-task Gaussian Processes. In *Advances in Neural Information Processing Systems 30 (NIPS 2017)*. Retrieved from https://papers.nips.cc/paper/6934-bayesian-inference-of-individualized-treatment-effects-using-multi-task-gaussian-processes

Arjas, E. (2014). Time to Consider Time, and Time to Predict? *Statistics in Biosciences*, *6*(2), 189–203. https://doi.org/10.1007/s12561-013-9101-1

Bica, I., Alaa, A. M., Jordon, J., & van der Schaar, M. (2020). Estimating Counterfactual Treatment Outcomes over Time Through Adversarially Balanced Representations. In *8th International Conference on Learning Representations (ICLR)*. Retrieved from https://openreview.net/pdf?id=BJg866NFvB

Brunner, F. J., Waldeyer, C., Ojeda, F., Salomaa, V., Kee, F., Sans, S., … Koenig, W. (2019). Application of non-HDL cholesterol for population-based cardiovascular risk stratification: results from the Multinational Cardiovascular Risk Consortium. *The Lancet*, *394*(10215), 2173–2183. https://doi.org/10.1016/S0140-6736(19)32519-X

Cai, T., Tian, L., Wong, P. H., & Wei, L. J. (2016). Analysis of randomized comparative clinical trial data for personalized treatment selections. *Biostatistics*, *17*(2), 249–263. https://doi.org/10.1093/BIOSTATISTICS

Candido dos Reis, F. J., Wishart, G. C., Dicks, E. M., Greenberg, D., Rashbass, J., Schmidt, M. K., … Pharoah, P. D. P. (2017). An updated PREDICT breast cancer prognostication and treatment benefit prediction model with independent validation. *Breast Cancer Research*, *19:58*(1). https://doi.org/10.1186/s13058-017-0852-3

Ference, B. A., Ginsberg, H. N., Graham, I., Ray, K. K., Packard, C. J., Bruckert, E., … Catapano, A. L. (2017). Low-density lipoproteins cause atherosclerotic cardiovascular disease. 1. Evidence from genetic, epidemiologic, and clinical studies. A consensus statement fromthe European Atherosclerosis Society Consensus Panel. *European Heart Journal*, *38*(32), 2459–2472. https://doi.org/10.1093/eurheartj/ehx144

Greenland, S. (2012). Causal Inference as a Prediction Problem: Assumptions, Identification and Evidence Synthesis. In *Causality: Wiley Series in Probability and Statistics* (pp. 43–58). Wiley Blackwell. https://doi.org/10.1002/9781119945710.ch5

Lamont, A., Lyons, M. D., Jaki, T., Stuart, E., Feaster, D. J., Tharmaratnam, K., … Van Horn, M. L. (2018). Identification of predicted individual treatment effects in randomized clinical trials. *Statistical Methods in Medical Research*, *27*(1), 142–157. https://doi.org/10.1177/0962280215623981

Li, J., Zhao, L., Tian, L., Cai, T., Claggett, B., Callegaro, A., … Wei, L.-J. (2016). A predictive enrichment procedure to identify potential responders to a new therapy for randomized, comparative controlled clinical studies. *Biometrics*, *72*(3), 877–887. https://doi.org/10.1111/biom.12461

Lim, B. (2018). Forecasting Treatment Responses Over Time Using Recurrent Marginal Structural Networks. In *32nd Conference on Neural Information Processing Systems (NeurIPS 2018)* (pp. 7494–7504). Retrieved from http://papers.nips.cc/paper/7977-forecasting-treatment-responses-over-time-using-recurrent-marginal-structural-networks

Nguyen, T.-L., Collins, G. S., Landais, P., & Le Manach, Y. (2020). Counterfactual Clinical Prediction Models Could help to Infer Individualised Treatment Effects in Randomised Controlled Trials – an Illustration with the International Stroke Trial. *Journal of Clinical Epidemiology*, *125*, 47–56. https://doi.org/10.1016/j.jclinepi.2020.05.022

Pajouheshnia, R., Damen, J. A. A. G., Groenwold, R. H. H., Moons, K. G. M., & Peelen, L. M. (2017). Treatment use in prognostic model research: a systematic review of cardiovascular prognostic studies. *Diagnostic and Prognostic Research*, *1*(1), 15. https://doi.org/10.1186/s41512-017-0015-0

Pearl, J. (2011). *Causality: Models, reasoning, and inference, second edition*. Cambridge University Press. https://doi.org/10.1017/CBO9780511803161

Rasmussen, C. E., & Williams, C. K. I. (2006). *Gaussian Processes for Machine Learning*. the MIT Press. Retrieved from www.GaussianProcess.org/gpml

Robins, J. (1999). Association, Causation, And Marginal Structural Models. *Synthese*, *121*(1/2), 151–179. https://doi.org/10.1023/A:1005285815569

Schulam, P., & Saria, S. (2017). Reliable Decision Support using Counterfactual Models. In *Advances in Neural Information Processing Systems 30 (NIPS 2017)* (pp. 1697–1708). Retrieved from https://papers.nips.cc/paper/6767-reliable-decision-support-using-counterfactual-models

Silva, R. (2016). Observational-Interventional Priors for Dose-Response Learning. Advances in Neural Information Processing Systems 29 (NIPS 2016). Retrieved from http://papers.neurips.cc/paper/6107-observational-interventional-priors-for-dose-response-learning

Soleimani, H., Subbaswamy, A., & Saria, S. (2017). Treatment-response models for counterfactual reasoning with continuous-time, continuous-valued interventions. In *the 33rd Conference on Uncertainty in Artificial Intelligence (UAI)*. Retrieved from http://auai.org/uai2017/proceedings/papers/266.pdf

Sperrin, M., Martin, G. P., Pate, A., Van Staa, T., Peek, N., & Buchan, I. (2018). Using marginal structural models to adjust for treatment drop-in when developing clinical prediction models. *Statistics in Medicine*, *37*(28), 4142–4154. https://doi.org/10.1002/sim.7913

van Amsterdam, W. A. C., Verhoeff, J. J. C., de Jong, P. A., Leiner, T., & Eijkemans, M. J. C. (2019). Eliminating biasing signals in lung cancer images for prognosis predictions with deep learning. *Npj Digital Medicine*, *2*(1), 1–6. https://doi.org/10.1038/s41746-019-0194-x

Xu, Y., Xu, Y., & Saria, S. (2016). A Bayesian Nonparametric Approach for Estimating Individualized Treatment-Response Curves. In F. Doshi-Velez, J. Fackler, D. Kale, B. Wallace, & J. Wiens (Eds.), *Proceedings of the 1st Machine Learning for Healthcare* (pp. 282–300). PMLR. Retrieved from http://proceedings.mlr.press/v56/Xu16.pdf
